# Genotypic differences in symbiotic nitrogen fixation ability and seed yield of climbing bean

**DOI:** 10.1007/s11104-018-3665-y

**Published:** 2018-05-09

**Authors:** Norma Barbosa, Elizabeth Portilla, Hector Fabio Buendia, Bodo Raatz, Stephen Beebe, Idupulapati Rao

**Affiliations:** 1 International Center for Tropical Agriculture (CIAT), Km 17 Recta Cali-Palmira, A.A, 6713 Cali, Colombia; 2 Present Address: Plant Polymer Research Unit, National Center for Agricultural Utilization Research, Agricultural Research Service, United States Department of Agriculture, 1815 North University Street, Peoria, IL 61604 USA

**Keywords:** Seed yield, ^15^N natural abundance, Nitrogen derived from atmosphere, Nitrogen derived from soil, Nitrogen use efficiency, Pod harvest index, Shoot biomass

## Abstract

**Aims:**

Symbiotic nitrogen fixation (SNF) contributes to improve grain yield under nitrogen (N) deficiency. Climbing beans are known to be superior to bush beans in their potential for SNF. The main objectives of this study were to: (i) quantify genotypic differences in SNF ability of climbing beans using ^15^N natural abundance method; (ii) identify climbing bean genotypes that combine high SNF ability with high yield potential that could serve as parents in the breeding program; and (iii) test whether δ^15^N in seed can be used instead of δ^15^N in shoot for estimating SNF ability.

**Methods:**

98 Climbing bean genotypes were evaluated for SNF ability in terms of nitrogen derived from the atmosphere (%Ndfa). Field trials were conducted at two locations in Colombia.

**Results:**

Significant genotypic differences were observed in SNF ability. Good yielding lines with 4.6 t ha^−1^ fixed as much as 60% of their N (up to 92 kg of N fixed ha^−1^) without application of N fertilizer to soil.

**Conclusions:**

Based on evaluations from both locations, seven climbing bean lines (ENF 235, ENF 234, ENF 28, ENF 21, MAC 27, CGA 10 and PO07AT49) were identified as promising genotypes. Seed samples can be used to determine SNF ability, to select for genotypes with superior SNF ability.

## Introduction

Common bean (*Phaseolus vulgaris* L.) is the most important food legume in the tropics of Latin America and eastern and southern Africa. It is an inexpensive source of protein and important for food security for small farmers in countries with endemic poverty (Rao 2014). This crop is grown by smallholder farmers mainly by women where it is often produced on marginal lands with minimum use of inputs and exposed to low nitrogen (N), phosphorus (P) deficiencies and drought that reduce yields (Beebe et al. 2008). However, legume crops are able to acquire their N from soil and also from symbiotic nitrogen fixation (SNF) that provide the alternative source of available N to the individual plant (Unkovich et al. 2008). In common bean around 60% of N is derived from soil (Ndfs) with 40% supplied through SNF process (Unkovich and Pate 2000; Peoples et al. 2009). Improving SNF potential serves not only as a low cost strategy to maintain productivity but also contributes to the possibility to successfully grow grain legume crops with minimal N fertilizers in soils with N deficiencies, thereby reducing both the cost of crop production and risk for hunger and malnutrition (Vance 2001; Mafongoya et al. 2009)

Most of the research on improving SNF in common bean has been conducted using bush bean genotypes. The estimated mean value of %N derived from the atmosphere (%Ndfa) for bush bean is 40%, this value is moderately in contrast with 68% and 75% of soybean and faba bean, respectively (Unkovich and Pate 2000; Herridge et al. 2008; Peoples et al. 2009). However, under optimal conditions in the field, common bean can derive up to 73% of total plant N through SNF and fix up to 125 kg N ha^−1^ depending on soil N availability, rhizobium strain and drought level while under controlled conditions (in the greenhouse and use of hydroponic system) %Ndfa values could be higher due to effective inoculation with rhizobium strain in the absence of N supply (Hardarson et al. 1993a; Kipe-Nolt and Giller 1993; Kipe-Nolt et al. 1993b; Giller 2001; Douxchamps et al. 2010; Kamfwa et al. 2015; Polania et al. 2016).

Several researchers found a negative exponential relationship between rate of applied N fertilizer and N_2_ fixation and strong reduction of SNF under drought conditions (Leidi and Rodríguez-Navarro 2000; Wanek and Arndt 2002; Salvagiotti et al. 2008; Ramaekers et al. 2013; Devi et al. 2013; Beebe et al. 2014; Burchill et al. 2014; Polania et al. 2016). However, climbing beans exhibited higher SNF ability than bush bean, with a mean % N derived from the atmosphere (%Ndfa) value of 60% which is greater than reported bush bean values (Unkovich and Pate 2000; Hardarson and Atkins 2003; Ramaekers et al. 2013). Additionally, climbing beans are also capable of 40% higher yield potential compared to bush beans and show positive association between the climbing growth habit and SNF ability (Graham and Rosas 1977; Bliss 1993; Kipe-Nolt and Giller 1993; Graham and Vance 2000). Among the few reports of SNF in climbing beans, cv. Caballero was found to be efficient in SNF with values greater than 50% Ndfa and 81 kg N ha^−1^ fixed without abiotic stress limitation (Manrique et al. 1993). Thus identifying parental climbing bean genotypes that combine superior SNF ability with greater seed yield and higher tolerance to drought stress could be an important breeding strategy to improve bean production under unfavorable climatic conditions in marginal areas where improving food and nutritional security is a major challenge.

Use of natural abundance of ^15^N is considered as one of the best methods to quantify SNF in terms of %Ndfa, because it allows the estimation of N fixation in almost any situation where both N-fixing and non N-fixing plants are present at the same location (Unkovich et al. 2008; Douxchamps et al. 2010). Also, it allows to separate %Ndfa from % N derived from the soil (%Ndfs) (Boddey et al. 2000). This method normally uses shoot tissue, however, the evaluation of shoot tissue means high labor costs for plant breeding programs that deal with large number of breeding lines. Also, utilization of shoot biomass samples is complex because destructive sampling of the plot requires processing of a large quantity of material. Polania et al. (2016) validated the use of seed samples to quantity %Ndfa and found significantly positive association between %Ndfa from shoot and %Ndfa from seed in bush beans based on the principle that common beans mobilize much of their N from vegetative structures to the seed. For breeding programs on climbing beans, the higher shoot biomass production per plant compared to bush beans makes it even more difficult to use ^15^N method to quantify SNF in shoot tissue. Thus there is a need to validate the use of seed samples for estimating genotypic differences in SNF ability.

The main objectives of this study were to: (i) quantify genotypic differences in SNF ability of climbing beans using ^15^N natural abundance method; (ii) identify genotypes that combine high SNF ability with high yield potential that could serve as parents in the breeding program; and (iii) test whether δ^15^N in seed can be used instead of δ^15^N in shoot for estimating SNF ability in climbing beans.

## Materials and methods

### Experimental site and meteorological conditions

**Fig. 1:**
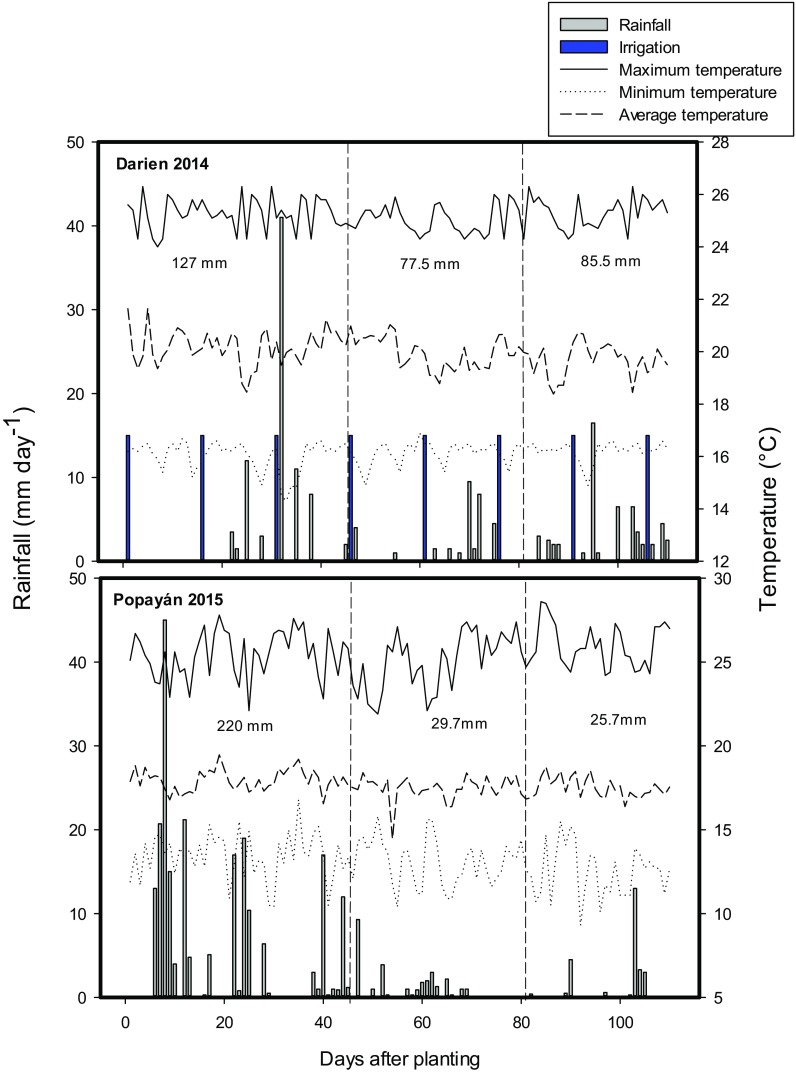
Rainfall, irrigation events and temperatures during crop growing period at Darien 2014 and Popayan 2015, Colombia

Field trials were conducted at two locations in Colombia: on a farm at Darien (June to September 2014) and at the CIAT Experimental Station at Popayan (June to September in 2015). Before establishing the field trials, soil chemical characteristics were determined for each field site by collecting 9 soil samples (0–20 cm soil depth) to represent spatial variability within the experimental area. The following characteristics were determined: soil organic matter (SOM) content, total N content, plant available N, available P concentration, and initial population and post-inoculation population of *Rhizobium* (cfu g^−1^ soil) using most probable number (MPN) technique (CGIAR and IRRI 1990). These data were used to map spatial variability in soil pH and soil P (Bray II extraction) availability (Bray and Kurtz 1945). Field site at Darien is located at 3°55′N and 76°28′W, with an altitude of 1600 m above sea level (masl). Field site at Popayán is located at 2°25′39″ N and 76°37′17″ W with an altitude of 1750 masl. The soil at both sites is an Inceptisol (Typic Dystrandept) and the differences in soil characteristics are shown in Table 1. The two locations differed mainly in altitude, soil nitrogen availability and rainfall distribution. Rainfall distribution, irrigation events and temperature changes during crop growing period at Darien and Popayan were recorded (Fig. 1). At Darien location, the total accumulated rainfall was 170 mm plus 120 mm of supplemental irrigation corresponding to a total water applied as 290 mm during crop growth. In contrast, at Popayan, total rainfall accumulation was 275 mm without any supplementary irrigation and 80% of total rainfall was noted before 40 days after sowing while the rest of growth cycle (80 days) received only 55.4 mm resulting in terminal drought stress during seed filling period.

**Table 1:** Soil chemical characteristics and population of Rhizobium at two locations in Colombia

Location	pH	SOM (g kg^−1^)	Total N (%)	Available N (kg ha^−1^)	Available P (mg/kg)	Population of *Rhizobium* before inoculation (cfug^−1^soil)	Population of *Rhizobium* after inoculation (cfug^−1^soil)
Darien	5.5	71	0.35	26.6	5.1	1.0 × 10^4^	6.0 × 10^7^
Popayan	5.7	144	0.7	72.4	4.9	5.0 × 10^4^	1.54 × 10^5^

SOM = soil organic matter

Both trials were established using 2800 m^2^ field area at each field site without application of N fertilizer. Other nutrients were applied (kg ha^−1^) as 40 P (as GranoFOS 40®), 30 K, 54 Ca, 25 S, 18 Mg and a mix of essential micronutrients (Microcoljab R). Fertilizer was applied at the time of sowing. Additionally, weeds were controlled by application of herbicides, pests and diseases were controlled by application of insecticides and fungicides, as needed. Total rainfall during the active crop growth was 290 mm in 2014 at Darien and 275 mm in 2015 at Popayan. The trial at Darien was supplemented with irrigation (120 mm) to minimize drought effects during seed filling period. But the trial at Popayan had limited rainfall during seed filling period (32.5 mm) which resulted in terminal type of drought stress (Fig. 1).

### Plant material and bacterial strain

The plant materials consisted of 100 bean genotypes, including 67 ENF lines, 13 CGA lines, 2 MBC lines, 9 MAC lines, and 9 further genotypes including checks GLORIABAMBA NN, D. MORENO, BOLIVAR and accessions G 19839, G 2333 and G 5702. Out of the 100 bean genotypes, 98 genotypes used were climbing beans. ENF lines were developed from intra- and inter-gene pool single and double crosses to combine resistance to different diseases. CGA lines have commercial grain type including major market classes of red, red mottled, cream mottled, white and yellow color. MBC lines referred to Mid-altitude BCMNV resistant climbing types. The checks included G5702 (super nodulating) and two parental lines G2333 (climbing bean) and G19839 (bush bean) that were evaluated previously by Ramaekers et al. (2013). Another important check was Gloriabamba non-nodulating (NN) genotype. The seven MAC lines referred to mid-altitude climbing lines MAC 4, MAC 9, MAC 33, MAC 74, MAC 56, MAC 25, MAC 57 that are currently grown in mid-altitude environments as climbing beans with high yield potential and good commercial grain type. Seed material was supplied by the Andean Bean Breeding team of CIAT, Cali, Colombia.

All genotypes were inoculated with *Rhizobium tropici* CIAT 899. The inoculum preparation was carried out following the methodology reported by Remans et al. (2008). Briefly, the strain was grown overnight in Liquid Yeast Mannitol agar medium at 30 ^°^C and at pH 6.8. These liquid cultures were washed twice with 10 mM MgSO_4_ and suspended in 10 mM MgSO_4_ at a density of 10^7^ colony forming units per ml (cfu/ml). For the field experiment, 30 ml of this inoculum was injected into sterilized humus in air-closed plastic bags (50 g humus per bag). The humus was thoroughly mixed and left at room temperature for 7 days (mixed once a day). The inoculation was carried out by soil drenching at 8 and 15 days after emergence (DAE) of the trials.

### Experimental design

Experiments at Darien and Popayán consisted of a 10 × 10 alpha-lattice design with three replications and with a total of 600 rows. The experimental units consisted of two rows of 2.5 m long with a row–to-row distance of 1.0 m and plant-to-plant spacing of 10 cm which is equivalent to 25 seeds per row.

### Determination of morpho-physiological traits and symbiotic nitrogen fixation ability (%Ndfa) using shoot and seed tissue

Out of the two rows planted for each genotype in each replication, one row was used for determining morpho-physiological traits and the other row was used for seed yield determination. Morpho-physiological traits were measured at three different growth stages of the crop: flowering, mid-pod fill and harvest. Phenology was monitored by recording days to flowering (number of days from sowing until 50% of plants with at least one open flower in a plot) and days to physiological maturity (number of days from sowing until at least 90% of the plants reach physiological maturity) as described by Ambachew et al. (2015). At each growth stage, a 0.5 m long row of six plants was harvested. At flowering growth stage (R6), genotypic differences in nodulation ability using three plants within a row were visually estimated using a scale of 1–9 where 1 was poor nodulation and 9 was high nodulation ability. This nodulation score was based on: estimation of number of nodules, nodule size, homogeneity of three roots, nodules abundance and viability (van Schoonhoven and Pastor-Corrales 1987).

Using six plants within a row (0.5 m), shoot biomass (SB) and dry matter distribution among plant parts (leaf and stem) were determined by drying the plant tissue in an oven at 60 °C for 2 days. At mid-pod fill growth stage (R7–8), SPAD chlorophyll meter readings (SCMR as SPAD units) were taken on fully expanded young leaves of 10 plants within a row using a SPAD chlorophyll meter (SPAD-502 Chlorophyll Meter, Minolta Camera Co., Ltd., Japan). At harvest time (R9), one full row was used for estimating seed yield (Yd) in kg ha^−1^ and 0.5 m length of a second row was used to determine yield components (pod number per plant, seed number per pod, seed and pod dry weight, 100 seed weight). Pod harvest index (PHI, seed dry weight at harvest/pod dry weight at harvest × 100), nitrogen use efficiency (NUE, kg of grain produced per kg of N in shoot biomass) and nitrogen partitioning index (NPI, kg of N in seed/kg of N in shoot biomass at mid-pod fill) were also determined.

The oven-dried samples of shoot and seed tissue were finely ground using a ball-mill and weighed using a microbalance (Sartorius Cubis® Germany) to pack 3.0 mg of each sample in a tin capsule. Samples were sent to University of California-Davis Stable Isotope Laboratory Facility in USA for ^15^N isotope analyses, δ^15^N and total N concentration (%) were measured using a PDZ Europa ANCA-GSL elemental analyzer interfaced to a PDZ Europa 20–20 isotope ratio mass spectrometer (Sercon Ltd., Cheshire, UK; Sharp 2005). %Ndfa_Sh and %Ndfa_Sd (N derived from atmosphere in shoot and seed) were calculated for each experimental unit following the equation given by Shearer and Kohl (1986):

$$ \% Ndfa=\frac{\ \delta 15\  of\ non\ fixing\kern0.5em plant-\delta 15\  of\ Nfixing\ legume}{\delta 15\  of\ Nfixing\ legume-\beta }\ x\ 100 $$

Where β is the δ^15^N value from the nitrogen-fixing genotype grown in N-free medium (sand culture). The B-value, i.e. the isotopic fractionation during N_2_ fixation, was obtained from a pot experiment in the greenhouse at the International Centre of Tropical Agriculture (CIAT), Colombia (3°29′N, 76°2′W), following the procedure of Unkovich et al. (2008). Seventy five (75) pots were filled with washed white quartz, planted with bean with different growth stages and habits corresponding to flowering, mid pod fill and harvest; the genotypes used were CAL 96 (habit I), SMC 140 (II), GGR18(III), MAC 33 (IV) and non nodulate control BAT 477 NN. Watered daily with an N-free nutrient solution Norris and Date 1976) containing (per liter of deionized water) KH_2_PO_4_ (0.27 g), K_2_SO_4_ (0.35 g), CaSO_4_·2H_2_O (1.0), MgSO_4_·7H_2_O (0.25 g), HBO_3_ (4.0 mg), MnCl 2·4H_2_O (0.99 mg), ZnSO_4_·7H_2_O (0.58 mg), CuSO_4_·5H_2_O (0.125 mg), FeCl_3_·6H_2_O (5.4 mg), and Na_2_MoO_4_·2H_2_O (0.1 mg). The inoculum used was CIAT 899 10 × 10^7^ UFC/ml. Shoot and grain δ^15^N values were corrected for seed N effect using a mass balance (Boddey et al. 2000; Nebiyu et al. 2014) .

To determine β we used MAC 33 to represent climbing beans. Different β values were used to determine Ndfa from shoot biomass and seed (Polania et al. 2016; Pacheco et al. 2017). We used the β-value of −3.32‰ for Ndfa_Sh at mid-pod fill growth stage and − 2.78 for Ndfa_Sd for the seed at harvest time.

Total *Ndfs* (N derived from soil, TNdfs) and Total *Ndfa* (N derived from atmosphere, TNdfa) per unit area were estimated using the following formulas:

$$ Total\ Ndfa\ in\  kg\ {ha}^{-1}= Total\ N\  in\ shoot\ or\ seed\ in\  kg\ {ha}^{-1}x\kern0.75em \% Ndfa\kern0.5em in\ shoot\ or\ seed/100 $$

$$ Total\ Ndfs\ in\  kg\ {ha}^{-1}= Total\ N\  in\ shoot\ or\ seed- Total\ Ndfa\ in\ shoot\ or\ seed\ in\  kg\ {ha}^{-1} $$

### Statistical analysis

All data were analyzed using the SAS (v 9.0) PROC MIXED and PROC CORR (SAS Institute Inc., 2008). The adjusted means for each genotype and the environment (Darien and Popayan) were obtained using the mixed models theory together with the MIXED procedure considering the effects of the replications and blocks within replications as random and genotypes as fixed. Correlation coefficients were calculated by the PROC CORR. Values marked with *, ** or *** are statistically significant at probability levels of 5%, 1% and 0.1%, respectively. The relationship between traits at the phenotypic level was assessed using PRINCOMP procedure for principal components analysis (PCA) (SAS Institute Inc., 2008).

## Results

### Genotypic differences in SNF ability in relation to environmental conditions

Environmental conditions can influence the SNF characteristics and this influence can vary among genotypes. Climbing bean lines showed genotypic differences in SNF ability in terms of %Ndfa under different soil and rainfall distribution conditions at Darien and Popayan (Table 2). Although both sites had similar total water supply during the growth season, at Popayan only 20% of total rainfall was observed beyond 40 days after sowing, leading to terminal drought stress during the seed filling period affecting SNF ability and seed yield (Fig. 1). In addition, the soil N availability at Popayan was higher than at Darien 72.4 and 26.6 kg ha^−1^, respectively, and this condition could suppress the SNF process (Table 1). Soil conditions such as soil organic matter content, soil pH and soil P availability were favorable at both locations. Based on the soil and climatic conditions, the Darien location can be considered more favorable than Popayan for the SNF process.

**Table 2:** Genotypic variation in morpho-physiological traits, seed yield and SNF characteristics of 100 genotypes of common bean grown at two locations (Darien and Popayan) in Colombia

Trait	Experiment	Mean	Min	Max	GxE	G	E	LSD 0.05
*Days to flowering* (DF)	Darien	46.5 ± 0.3	41.0	69.0	**	***	***	a
	Popayan	48.2 ± 0.3	42.0	65.0				b
*SPAD Chlorophyll meter readings* (SCMR)	Darien	38.4 ± 0.3	33.7	43.9	**	***	***	a
	Popayan	42.5 ± 0.3	37.8	47.7				b
*Nodule score (1–9)* (NENCB*)*	Darien	7.0 ± 0.95	1.0	8.0	***	***	***	a
	Popayan	3.0 ± 0.7	1.0	7.0				b
*Shoot biomass (kg ha*^*−1*^*)* (SB)	Darien	3753 ± 285.3	1527	7488	***	ns	***	a
	Popayan	5594 ± 285.3	2901	8876				b
*Pod Harvest index (%)* (PHI)	Darien	74.7 ± 0.2	65.2	83.5	**	***	***	a
	Popayan	70.3 ± 0.2	57.3	78.2				b
*Seed yield (kg ha*^−1^) (Yd)	Darien	3592 ± 157.1	786	6830	***	**	***	a
	Popayan	3172 ± 156.9	1931	4644				b
*% N shoot* (%N_Sh)	Darien	3.9 ± 0.17	2.90	4.60	***	***	***	a
	Popayan	4.4 ± 0.13	3.60	5.04				b
*% N seed* (%N_Sd)	Darien	3.7 ± 0.17	3.01	4.70	ns	ns	ns	a
	Popayan	3.8 ± 0.17	3.10	4.50				a
*N total in shoot (kg N ha*^*−1*^*)* (TN_Sh)	Darien	148.1 ± 13.4	59.0	295.7	***	ns	***	a
	Popayan	251.2 ± 13.5	131.5	401.8				b
*N total in seed (kg N ha*^*−1*^*)* (TN_Sd)	Darien	130.1 ± 8.0	60.4	200.5	ns	**	ns	a
	Popayan	120.5 ± 8.0	73.7	183.1				a
*%Ndfa_Shoot* (%Ndfa_Sh)	Darien	24.6 ± 5.5	0.0	44.6	***	***	***	a
	Popayan	19.1 ± 3.1	0.0	32.6				b
*%Ndfa_seed* (%Ndfa_Sd)	Darien	41.3 ± 6.6	0.0	67.8	***	***	ns	a
	Popayan	34.4 ± 2.8	0.2	43.7				b
*Total Ndfa shoot (kg N ha*^*−1*^*)* (TNdfa_Sh)	Darien	35.1 ± 4.4	1.8	85.8	**	***	***	a
	Popayan	45.2 ± 4.4	0.1	93.4				b
*Total Ndfa seed (kg N ha*^*−1*^*)* (TNdfa_Sd)	Darien	49.2 ± 2.0	0.2	91.2	***	***	Ns	a
	Popayan	42.4 ± 2.1	0.3	60.1				b
*Total Ndfs shoot (kg N ha*^*−1*^*)* (TNdfs_Sh)	Darien	113.1 ± 12.0	48.1	265.0	**	ns	***	a
	Popayan	205.9 ± 12.0	108.9	340.4				b
*Total Ndfs seed (kg N ha* ^−1^) (TNdfs_Sd)	Darien	81.7 ± 6.7	35.5	144.5	ns	*	Ns	a
	Popayan	78.1 ± 6.8	47.7	181.9				a
*Nitrogen Partition index (%)* (NPI)	Darien	102.3 ± 4.9	45.4	215.3	***	***	***	a
	Popayan	53.9 ± 5.0	25.4	94.2				b
*Nitrogen Use efficiency (kg seed kg*^*−1*^ *N)* (NUE)	Darien	27.8 ± 1.3	11.4	60.7	***	***	***	a
	Popayan	14.2 ± 1.3	6.5	23.5				b

Significance indicates differences between locations (LSD 0.05). Significance levels of interactions between genotypes (G) and environments (E) and interactions (GxE) are also shown

*,**,*** Significant difference at 0.05, 0.01 and 0.001 probability levels; ± refers to standard error. Different letters for LSD indicate significant differences between locations as estimated from PROCMIXED procedure

### Genotypic differences in morpho-physiological traits, seed yield and SNF characteristics

Genotypes could vary in their SNF ability measured in terms of Ndfa_Sh or Ndfa_Sd and these differences can influence seed yield. Results on genotypic variation in morpho-physiological traits, seed yield, N efficiency traits and SNF characteristics of 100 genotypes of common bean grown at two locations are shown in Table 2. Genotypic differences in SNF traits showed that the values of SNF ability were greater at Darien than at Popayan location (41.3 and 34.4 %Ndfa_Sd, respectively) with significant differences (*p* < 0.05). Also the %Ndfa_Sh values were higher at Darien than at Popayan corresponding to 24.6 and 19.1% (*p* < 0.05), respectively. Mean nodule score of the 100 genotypes was also higher at Darien than at Popayan based on nodule number, viability and size. (Table 2).

The genotype effects and the GxE interaction effects were significant (*p* < 0.05) for majority of morpho-physiological traits (Table 2). In contrast, few traits such as TN_Sd, TNdfs_Sd and pod harvest index were not affected by the environment (location). Values of both shoot biomass accumulation and TN_Sh were higher at Popayan showing greater N accumulation in shoot biomass together with lower level of N partitioning to seed (lower values of NPI) (Table 2).

**Fig. 2:**
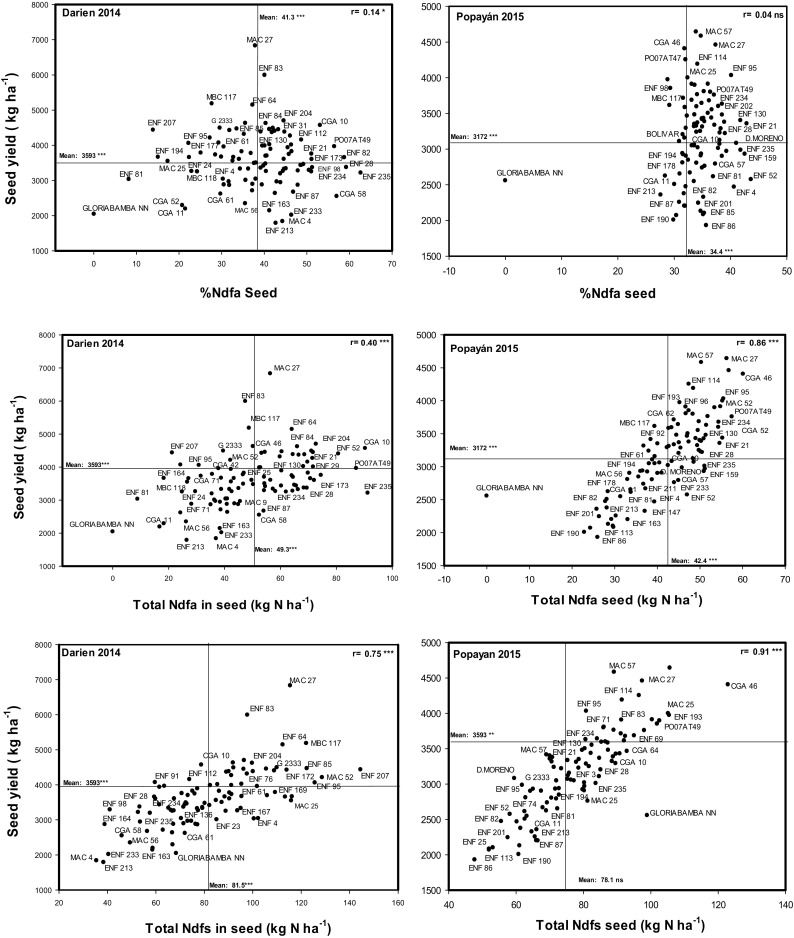
Identification of genotypes that combine high values of % N derived from atmosphere (%Ndfa), total N derived from atmosphere (TNdfa_Sd) and total N derived from soil (TNdfs_Sd) with seed yield grown at Darien in 2014 and at Popayan in 2015. The value of r represents Pearson correlation coefficient, and genotypes with higher SNF and seed yield were identified in the upper, right hand quadrant

Evaluating the relationships between seed yield and SNF ability among the genotypes could help to identify promising genotypes that combine greater ability in SNF with higher values of seed yield. Relationships between seed yield and SNF ability in terms of %Ndfa_Sd, TNdfa and TNdfs were positive and significant at Darien. The correlation coefficient between seed yield and %Ndfa_Sd at Darien was small but significant (*r* = 0.14*) (Fig. 2). In contrast at Popayan where the conditions for SNF were unfavorable, the correlation between seed yield and %Ndfa_Sd was not significant. Several genotypes were identified that combine greater values of seed yield above 3000 kg ha^−1^ with moderate to high values of %Ndfa and TNdfa at both locations (Table 3). Seven lines ENF 235, ENF 28, PO07AT49, ENF 234, ENF 21, CGA 10 and MAC 27 were promising in their SNF ability by maintaining higher values of %Ndfa and seed yield (Table 3). MAC 27 was among the high yielding genotypes and this line also showed greater SNF ability in terms of %Ndfa_Sd and moderate values of %Ndfa_Sh (Table 3). The non-nodulating Gloriabamba genotype and some genotypes identified as non-promising such as ENF 81, CGA 11 and ENF 213, showed lower values of seed yields together with lower values of %Ndfa at both locations (Table 3). Nevertheless, these genotypes showed greater values of TNdfs at Popayan (Table 2) where the available N in soil was higher (Table 1). The highest seed yield correlations were observed with TNdfs and TN_Sd, but the former were more positive at Popayan (*r* = 0.91***) than at Darien (*r* = 0.75***). In contrast, TNdfs_Sd values showed significantly negative correlations with %Ndfa_Sh and %Ndfa_Sd, more strongly at Darien with *r* = −0.67*** and − 0.34 *** for Popayan, respectively, showing the negative influence of high soil N availability on SNF ability (Table. 4).

**Table 3:** Genotypic differences in % N derived from atmosphere in shoot tissue (%Ndfa_Sh), % N derived from the atmosphere in seed tissue (%Ndfa_Sd), of a subset of high, moderate and low SNF ability genotypes (Mean values from two locations)

	%Ndfa_Sd	%Ndfa_Sh	seed yield (kg ha^−1^)	LSD 0.05
High SNF ability				
ENF 235	54	28	3096	a
ENF 28	51	18	3296	ab
PO07AT49	49	20	3863	ab
ENF 82	48	31	3062	ab
ENF 234	47	25	3436	abc
ENF 21	46	31	3811	abc
ENF 112	46	23	3690	abc
ENF 159	46	38	3127	abc
CGA 64	45	18	3399	abc
ENF 52	45	32	3487	abc
ENF 164	45	32	2948	abc
CGA 10	44	22	3933	bc
Moderate SNF ability				
CGA 56	40	23	3478	bc
ENF 96	40	26	3748	bc
ENF 182	40	23	3863	bc
ENF 204	40	20	4141	bc
ENF 84	40	31	4126	bc
ENF 136	40	24	3164	bc
ENF 171	39	20	2962	bcd
MAC 27	39	19	5644	bcd
ENF 211	39	33	3072	bcd
ENF 188	39	14	3762	bcd
ENF 114	38	24	3528	cde
low SNF ability				
ENF 24	30	23	3315	feg
CGA 52	29	15	2988	feg
CGA 11	26	18	2347	fg
MAC 25	26	20	3770	fg
ENF 213	24	15	2068	fg
ENF 207	24	18	3880	fg
ENF 81	23	23	2822	fgh
GLORIABAMBA NN	0	0	2300	h
Mean	38.1	22.8	3462	
Sig. diff.	***	***	***	

*,**,***Significant difference at 0.05, 0.01 and 0.001 probability levels, respectively. Different letters for LSD indicate significant differences between genotypes as estimated from PROCMIXED procedure

**Table 4:** Pearson correlation coefficients between SNF traits, seed yield and other traits on a population of 100 common bean genotypes grown at Darien and Popayan, Colombia during 2014–2015

Darien										
Trait	% Ndfa_Sh	%Ndfa_Sd	TNdfa_Sd	TNdfa_Sh	TNdfs_Sd	TN_Sd	NUE	PHI	SB	Yd
% Ndfa_Sh	1									
%Ndfa_Sd	0.42***	1								
TNdfa_Sd	0.21**	0.72***	1							
TNdfa_Sh	0.76***	0.29**	0.30**	1						
TNdfs_Sd	−0.34***	−0.67***	0.07	0.10	1					
TN_Sd	−0.16*	0.15*	0.52***	0.09	0.81***	1				
NUE	0.11	0.12*	0.12*	0.39**	0.33**	0.35**	1			
PHI	0.08	0.09	0.17*	0.16*	0.09	0.18*	0.02	1		
SB	0.05	0.04	0.21**	0.48***	0.28**	0.36**	−0.59***	0.14*	1	
Yd	0.14*	0.14*	0.40**	0.06	0.75***	0.94***	0.33**	0.24**	0.31	1
Popayán										
Trait	% Ndfa_Sh	%Ndfa_Sd	TNdfa_Sd	TNdfa_Sh	TNdfs_Sd	TN_Sd	NUE	PHI	SB	Yd
% Ndfa_Sh	1									
%Ndfa_Sd	0.46***	1								
TNdfa_Sd	0.02	0.35***	1							
TNdfa_Sh	0.58***	0.20**	0.24**	1						
TNdfs_Sd	−0.32**	−0.34***	0.73***	0.09	1					
TN_Sd	−0.23**	−0.09	0.89***	0.15*	0.96***	1				
NUE	0.02	0.03	0.31***	0.45***	0.31***	0.33***	1			
PHI	−0.21*	−0.05	−0.08	0.02	0.10*	0.10*	−0.05	1		
SB	0.20*	−0.07	0.32***	0.60***	0.37***	0.37***	−0.60***	0.10*	1	
Yd	0.21	0.04	0.86***	0.20**	0.91***	0.95***	0.33***	0.15*	0.41***	1

*,**,*** Significant difference at 0.05, 0.01 and 0.001 probability levels, respectively as estimated from CORR procedure

**Fig. 3:**
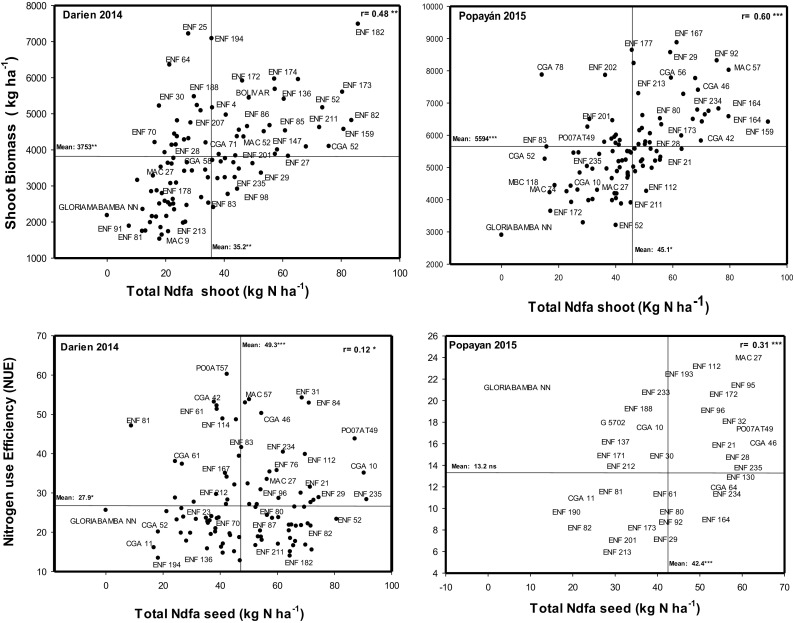
Identification of genotypes that combine high total N derived from atmosphere (TNdfa) with N use efficiency (NUE) and shoot biomass grown at Darien 2014 and at Popayán 2015. Genotypes with higher values of shoot biomass -TNdfa_Sh and NUE –TNdfa_Sd were identified in the upper, right hand quadrant

Genotypes could vary not only in their SNF ability but also in their ability to use N for the production of seed (NUE). Relationships of TNdfa_Sh with shoot biomass and NUE at two locations are shown in Fig. 3. Shoot biomass production values showed significantly lower correlation with TNdfa_Sh values at Darien (*r* = 0.48**) than at Popayan (*r* = 0.60***). At Popayan, ENF 167 was outstanding in its shoot biomass production while ENF 159 was outstanding for TNdfa_Sh (Fig. 3). Higher values of TNdfa_Sh at Darien were observed with ENF 182 and ENF 173. Relationship between NUE and TNdfa_Sh was positive and significant at Darien (*r* = 0.12*) and at Popayan *r* = 0.31**. The highest NUE value was observed at Darien with PO 07A T57 while MAC 27 was superior at Popayan (Fig. 3).

The values of NUE in terms of seed yield per unit of N uptake were 51% higher at Darien than at Popayan. In the same sense, the extent of N remobilized from shoot to seed (NPI) was 53% higher, similarly, pod harvest index which reflects the extent of photosynthate mobilization from pod wall to seed (Rao et al. 2013) was also 6% higher at Darien. This greater ability to mobilize photosynthates to seed at Darien correlated positively (*r* = 0.24**) with greater mean value of seed yield observed at Darien (Table 4). In accordance, at Popayan seed yield was decreased by 12% compared to Darien (Table. 2) and SNF traits values such as nodulation score, as % Ndfa and TNdfa showed a decrease of 7% and 14%, respectively (Table 2). Taken together, higher yields at Darien are the result of less investment in biomass and more efficient mobilization of N and C to seed.

The values of NUE at Darien showed a highly significant (*p* < 0.001) correlation with SNF traits corresponding to %Ndfa_Sh, %Ndfa_Sd, TNdfa_Sh, TNdfa_Sd (Table 4). In contrast, at Popayan no correlation was observed between %Ndfa and NUE. However, the genotypes ENF 235, ENF 21, CGA 10, PO07AT49 and MAC 27 were superior in combining higher values of NUE with greater values of TNdfa at both locations (Fig. 3).

### Principal component analysis

The relative contribution of different plant traits to SNF ability or seed yield can be evaluated using PC analysis. Since the PC analysis showed no significant difference between the two locations (Darien and Popayan) we have conducted combined analysis using data from both locations (Table 5). Seed yield was primarily associated with N availability from soil (TNdfs_Sh) and also to %Ndfa. The capacity to partition N to seed (NPI) and efficient use of N in seed production (NUE) showed good relationship (Table 5). Multivariate analysis of the results from both locations showed that the first three components of PC analysis could explain 83% of the variability observed in the SNF characteristics of 100 genotypes evaluated at two locations (Table 5). In component 1, the plant traits with the largest contribution to variability were: TN_Sh, TNdfs_Sh, %Ndfa_Sd, NUE and NPI. In component 2, the traits with the largest contribution to variability were: shoot biomass, TN_Sd, TNdfs_Sd and seed yield. In component 3, SNF characteristics such as nodulation score, TNdfa_Sd and TNdfa-Sh had larger contribution (Table 5).

**Table 5:** Eigen values, per cent of cumulative variance and component matrix for the principal component axes. Results presented are from both locations

Principals components	1	2	3	4	5	6
Eigenvalues % cumulative variance	4.230	3.360	1.553	0.639	0.503	0.392
	0.385	0.690	0.831	0.889	0.935	0.971
Component matrix						
NENCB	0.064	0.134	0.620	−0.611	0.140	0.444
SB	0.416	0.240	−0.106	−0.042	0.208	−0.097
TN_Sd	−0.154	0.497	0.004	−0.024	−0.334	−0.036
TN_Sh	0.413	0.254	−0.116	0.039	0.255	0.019
%Ndfa_Sd	0.314	0.302	0.218	0.047	−0.262	−0.663
TNdfa_Sh	0.276	0.153	0.350	0.729	0.052	0.415
TNdfs_Sh	0.375	0.234	−0.281	−0.245	0.294	−0.159
TNdfa_Sd	−0.067	0.348	0.507	0.075	−0.115	−0.639
TNdfs_Sd	−0.166	0.406	−0.345	−0.082	−0.343	0.424
Yd	−0.171	0.472	−0.099	0.055	0.036	−0.021
NUE	0.413	0.139	−0.062	0.106	0.502	0.082
NPI	0.424	0.090	0.009	0.067	0.537	−0.004
PHI	0.044	0.134	0.411	−0.630	0.120	0.243

### Comparison of two methods to estimate %Ndfa

**Fig. 4:**
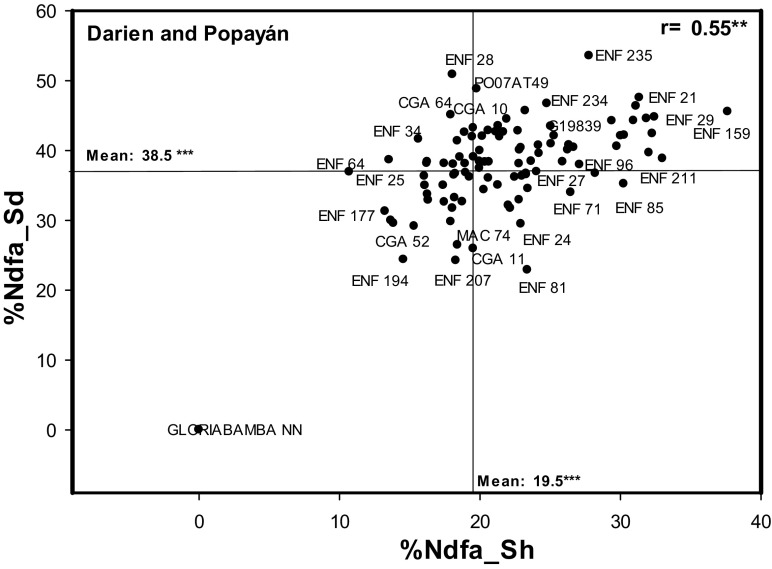
The relationship between %Ndfa between locations. Correlation of mean values of %Ndfa from seed (%Ndfa_Sd) and %Ndfa from shoot (%Ndfa_Sh) of 100 genotypes from both locations Darien and Popayán

We tested the relationship between %Ndfa_Sd measured using harvested seeds and %Ndfa_Sh measured at mid-pod filling growth stage at both locations. We found that individually, the correlations were both positive and highly significant for each location (Darien, *r* = 0. 42***; Popayan, *r* = 0.46***) (Table 4). We found an even stronger relationship between the Darien-Popayan mean values of %Ndfa_Sh and %Ndfa_sd (*r* = 0.55 ***). Additionally, we also found that the values of Ndfa_Sd were greater than those of Ndfa_Sh. For promising genotypes such as PO07AT49 and ENF 235 the %Ndfa value between shoot and seed differed by 24%. In contrast, a non promising genotype ENF 194 showed the values of Ndfa_Sd as 21% and Ndfa_Sh only 15% (Fig. 4).

## Discussion

### Genotypic differences in SNF ability

This study enabled to quantify genotypic differences in SNF ability in terms of %Ndfa and TNdfa in climbing bean and identified promising lines that combine high values of seed yield with SNF ability in an elite set of breeding lines (ENF). These lines were developed for improving seed yield and resistance to diseases such as anthracnose and bean common mosaic virus, which are important traits for bean improvement (Beebe 2012). This is the first report on the elite ENF climbing bean lines, quantifying the genotypic differences in SNF ability.

Farmers require high yielding plants in low input farming systems. High %Ndfa in combination with high seed yield is a way to produce high yields in low N fields without high fertilizer inputs. Furthermore, as most shoot biomass is used as animal fodder or incorporated in soil, high TNdfa referring to kg N fixed, helps farmers to produce N with lower inputs. The efficiency of major food crops in the recovery of applied N is often not more than 30% (Subbarao et al. 2013) and 60 kg N fixed through SNF would therefore be comparable to about 160 kg N applied as chemical fertilizer (Polania et al. 2016).

Significant genotypic variation in SNF ability and seed yield was reported using bush bean genotypes (Hardarson et al. 1993b; Kipe-Nolt and Giller 1993; Kipe-Nolt et al. 1993; Manrique et al. 1993; Douxchamps et al. 2010; Kamfwa et al. 2015; Polania et al. 2016). Results from this study on climbing beans support previous work which showed that climbing beans tend to have higher SNF potential as well as higher yield potential compared to bush beans, with a potential for SNF ability of over 90 kg N ha^−1^ (Giller 2001).

The process of SNF is affected by multiple stress factors, such as drought (Devi et al. 2013; Polania et al. 2016), low P availability in soil (Leidi and Rodríguez-Navarro 2000; Olivera et al. 2004; Ojiem 2006) and high nitrate or ammonium availability in soil that strongly suppresses SNF process (Giller 2001; Wanek and Arndt 2002; Salvagiotti et al. 2008; Burchill et al. 2014). Field evaluation at Popayan with soil having high SOM and high N availability together with terminal drought stress condition reduced the %Ndfa, TNdfa, NUE and seed yield. Our results are consistent with previous reports. Additionally, under higher N availability, the TNdfs was strongly correlated with TNdfa in spite of the positive effect of TNdfa on seed yield. These results indicate that TNdfs was a major contributor to plant N supply and seed yield formation for common bean. Thus simultaneous improvement for SNF ability and seed yield through breeding may impose some limitations to breeding efforts for some genotypes. Our results confirm previously reported detrimental factors to SNF and also indicate that there are marked differences in SNF ability under unfavorable conditions such as higher N availability combined with drought stress in soil (Giller 2001; Wanek and Arndt 2002; Salvagiotti et al. 2008; Burchill et al. 2014). In spite of this negative effect, there were promising genotypes that have shown less sensitivity to this condition, maintaining good values of %Ndfa and TNdfa across both field sites.

Our results have shown that the ability to remobilize and utilize N (NUE and NPI) was decreased by higher level of N availability and drought stress at Popayan where the mean value of NPI to seed was markedly reduced by about 53% and NUE by 48%.

The lower values of %Ndfa_Sd of climbing bean lines observed at Popayan were associated with higher values of SCMR, shoot biomass and also TN_Sh at mid-pod filling growth stage compared to the values observed at Darien. It is known that greater level of N accumulation in leaves and shoot tissue could inhibit SNF process by Autoregulation of Nodulation AON (Vadez and Sinclair 2001; Purcell et al. 2004; King and Purcell 2005). Using common bean, Coleto et al. (2014) showed that the variability in drought-induced inhibition of SNF was associated with accumulation of N in shoot tissue but not in nodules. This is in accordance with the increased accumulation contrasted by reduced remobilization of N from vegetative structures to seed (NUE and NPI) at Popayan.

Common bean lines can remobilize about 80% to 93% of its total N in the shoot to the seed under optimal conditions for SNF process (Ramaekers et al. 2013). Also, it has been shown that common bean accumulates preferentially the atmospherically fixed N into the seed (Westermann et al. 1985; Dubois and Burris 1986; Wolyn et al. 1991). Our results confirm these previous reports since the values of %Ndfa_Sd were 16% higher than those of %Ndfa_Sh at both locations.

At Darien, a few lines were very efficient in their SNF ability fixing about 90 kg N ha^−1^ based on TNdfa_Sd and TNdfa_Sh which is equivalent to 300 kg of applied fertilizer at 30% fertilizer efficiency while the best lines at Popayan fixed only up to 60 kg N ha^−1^. Vigorous plants permit higher levels of N accumulation to seeds if the plants could maintain greater ability to remobilize photosynthates (Polania et al. 2016). The high correlation between TNdfa_Sd and TNdfs_Sd at Popayan suggest low variability between the two uptake processes, these traits are driven by variability in seed. In contrast this correlation was not observed at Darien where the lines were more efficient and showed greater variability in SNF ability. Hence, TNdfa performance at low soil N availability could be increased genetically by breeding.

### Identification of promising lines with greater seed N and SNF ability under stress

Seven climbing bean genotypes (ENF 235, PO07AT49, CGA 10, ENF 28, ENF 21, ENF 234, MAC 27) reduced their SNF ability by only about 15% under high N availability and drought stress and these were considered as promising genotypes. This is because they were also superior in SNF ability and seed yield at Darien under low N availability. We found that the relationship between %Ndfa_Sd at Darien and %Ndfa_Sd at Popayan was positive and significant and these results are consistent with the results obtained by Polania et al. (2016) where the trait of %Ndfa_Sd showed positive and significant correlation between irrigated and drought stress conditions. Taken together, these results indicate that %Ndfa trait could be considered as stable across environments.

### Validation of the use of ^15^N natural abundance in seed as a method to quantify genotypic differences in SNF ability in climbing bean

This study allowed not only to quantify genotypic differences in climbing bean for SNF ability, but also to compare the estimation of %Ndfa using seed tissue (%Ndfa_Sd) vs. shoot tissue (%Ndfa_Sh). We used the β values of mid-pod filling growth stage for shoot biomass and harvest time for seed since it was shown from recent studies that β values are influenced by plant development in common bean (Polania et al. 2016; Pacheco et al. 2017). Past publications often used SNF in biomass at mid-pod fill, which is more representative for the whole N fixed, %TNdfa-Sh has often been used to infer desirable breeding traits in seed. %Ndfa_Sh is relevant for farming systems where biomass is used as animal food and/or fixed N remaining in soil improves soil quality. We consider that it is possible to replace measurement of %Ndfa_Sh with %Ndfa_Sd. We recommend the use of seed tissue since %Ndfa_Sd is more informative about protein for consumption in combination with TN_Sd. Recently, Polania et al. (2016) validated the usefulness of %Ndfa_Sd as a selection method to quantify phenotypic differences among bush bean lines grown under irrigated and drought stress conditions. Kamfwa et al. (2015) also used both shoot and seed samples to quantify SNF ability and suggested that selection for high SNF ability based on seed tissue analysis could be easier to integrate into most bean breeding programs. Similar results for %Ndfa using the ^15^N natural abundance technique in seed and the whole shoot have also been reported for other legume species (Bergersen et al. 1985).

We tested whether %Ndfa_Sd could be a useful method for selecting genotypes with superior SNF ability in on-going breeding programs. Results from two locations estimated under field conditions from shoot tissue at mid-pod filling growth stage (%Ndfa_Sh) and using seed tissue at harvest (%Ndfa_Sd), showed that Ndfa values from seed are suitable to quantify genotypic differences in SNF ability. The highly significant and positive correlation (*r* = 0.55***) observed between both methods using data from both locations validates the usefulness of %Ndfa_Sd as a selection method for %Ndfa_Sh. The use of seed tissue to estimate SNF ability could be implemented in on-going breeding programs to reduce costs in collecting and analyzing shoot tissue. Several bean breeding programs working on biofortified beans are aiming to increase iron (Fe) and zinc (Zn) levels in the seed (Blair et al. 2011). These programs currently use finely ground seed tissue to estimate Fe and Zn concentration in grain. The same ground tissue can be used to estimate SNF ability, lowering costs and processing time, so that improved bean lines that combine more desirable traits can be developed.

## Conclusions

Significant genotypic differences were observed in SNF ability among climbing beans at two field locations. %Ndfa and seed yield values were higher at Darien (site with low available N in soil) than at Popayan (site with higher available N in soil with terminal drought stress) and we found a positive association between SNF ability and seed yield. The line MAC 27 was outstanding in SNF ability and seed yield while seven genotypes (ENF 235, ENF 234, ENF 28, ENF 21, PO 07AT49, CGA 10) also exhibited superior performance. These promising lines also combined other desirable plant traits such as greater level of shoot biomass production, total N uptake in shoot biomass, and greater ability to partition a higher proportion of N to seed. These lines could serve as potential parents for breeding programs aiming to combine SNF ability and yield potential with other desirable traits like biotic stress resistance. In addition, this study validated the usefulness of using seed tissue to estimate SNF ability of climbing beans. This methodology could be implemented in on-going breeding programs to reduce costs in collecting and analyzing shoot tissue.

## Abbreviations

DF: Days to flowering
DPM: Days to physiological maturity
TNdfs: Nitrogen derived from soil per hectare
TNdfa: Nitrogen derived from the atmosphere per hectare
%Ndfa_Sd: Percentage of nitrogen derived from the atmosphere using seed
%Ndfa_Sh: Percentage of nitrogen derived from the atmosphere using shoot
NENCB: Nodule score (1–9)
%N_Sh: Nitrogen content in shoot in per cent
%N_Sd: Nitrogen content in seed in per cent
TN_Sh: Total nitrogen in shoot per hectare
TN_Sd: Total nitrogen in seed per hectare
SB: Shoot biomass at mid-pod filling
SOM: Soil organic matter
SCMR: SPAD chlorophyll meter reading
Yd: Seed yield per hectare
PHI: Pod harvest index
NPI: Nitrogen partitioning index
NUE: Nitrogen use efficiency
